# *Escherichia coli* infection indicates favorable outcomes in patients with infected pancreatic necrosis

**DOI:** 10.3389/fcimb.2023.1107326

**Published:** 2023-03-27

**Authors:** Haosu Huang, Jie Peng, Caihong Ning, Qin Wei, Jiarong Li, Chiayen Lin, Zefang Sun, Lu Chen, Shuai Zhu, Dingcheng Shen, Gengwen Huang

**Affiliations:** ^1^ Department of Gastroenterology, Xiangya Hospital, Central South University, Changsha, Hunan, China; ^2^ National Clinical Research Center for Geriatric Disorders, Xiangya Hospital, Central South University, Changsha, China; ^3^ Department of Pancreatic Surgery, General Surgery, Xiangya Hospital, Central South University, Changsha, Hunan, China; ^4^ Department of Hernia and Abdominal Wall Surgery, Xiangya Hospital, Central South University, Changsha, Hunan, China

**Keywords:** infected pancreatic necrosis, *Escherichia coli*, acute pancreatitis, outcomes, mortality

## Abstract

**Introduction:**

Infected pancreatic necrosis (IPN) is a severe complication of acute necrotizing pancreatitis with increasing morbidity. *Escherichia coli* is the most frequently cultured microorganism in IPN. However, the implications of *Escherichia coli* infection on the outcomes of patients with IPN remain unclear. Therefore, this study aimed to evaluate the clinical impacts of *Escherichia coli* infection on IPN.

**Methods:**

A prospective database with consecutive patients with IPN between January 2010 and April 2022 at a tertiary hospital was *post-hoc* analyzed. The clinical and microbiological characteristics, surgical management, and follow-up data of patients with and without *Escherichia coli* infection were compared.

**Results:**

A total of 294 IPN patients were enrolled in this cohort. Compared with non-*Escherichia coli* infection cases (n=80, 27.2%), patients with *Escherichia coli* infection (n=214, 72.8%) were characterized by more frequent polymicrobial infections (77.5% vs. 65.0%, *P*=0.04) but a lower occurrence of severe acute pancreatitis (SAP) (42.5% vs. 61.7%, *P*=0.003). In addition, significantly lower mortality (12.5% vs. 30.4%, *p*=0.002), fewer step-up surgical interventions (73.8% vs. 85.1%, *P*=0.025), and a lower rate of multiple organ failure (MOF) (25.0% vs. 40.2%, *P*=0.016) were also observed in patients with *Escherichia coli* infection. Multivariate analysis of mortality predictors indicated that MOF (odds ratio [OR], 6.197; 95% confidence interval [CI], 2.373–16.187; *P*<0.001) and hemorrhage (OR, 3.485; 95% CI, 1.623–7.487; *P*=0.001) were independent predictors associated with higher mortality in patients with IPN. *Escherichia coli* infection was significantly associated with a lower mortality (OR, 0.302; 95% CI, 0.121–0.751; *P*= 0.01).

**Conclusion:**

*Escherichia coli* infection indicates a favorable prognosis in patients with IPN, although the mechanism needs further investigation.

## Introduction

Acute pancreatitis (AP) is an inflammatory disorder of the pancreas that can cause severe local and systemic complications (Boxhoorn et al., 2020). Approximately 15–20% of patients with AP develop acute necrotizing pancreatitis (ANP) (Iannuzzi et al., 2022). One of the most common causes of death in patients with AP is infected pancreatic necrosis (IPN), a severe complication of ANP with a mortality of 20–30% (Baron et al., 2020). Previous studies have reported the microbiological profile of organisms in IPN. However, the results were not entirely consistent among studies due to different hospitals, clinical practices, and practices of antibiotic usage (Garret et al., 2020; Jiang et al., 2020; Wolbrink et al., 2020). In a retrospective study, Mowbray et al. compared their findings with eight other studies evaluating pathogens that caused IPN (Mowbray et al., 2018). They revealed a predominance of gut bacteria in IPN, including *Escherichia coli* (20%), which was also confirmed in our previous studies (24-29%) (Ning et al., 2019; Li et al., 2022). Notably, *Escherichia coli* was one of the most frequently cultured microorganisms in IPN.

Recently, studies have reported that several pathogenic microorganisms were identified as independent risk factors for increased mortality in IPN, including fungal infection, Carbapenem-resistant *Klebsiella pneumoniae* and *Acinetobacter baumanii* infection as well as candidemia and multidrug-resistant organisms (MDROs) infection (Moka et al., 2018; Rasch et al., 2018; Ning et al., 2019; Würstle et al., 2019; Moran et al., 2022). However, as one of the most frequently cultured microorganisms in IPN, *Escherichia coli* has never been investigated for its impact on the prognosis of patients with IPN. Therefore, we conducted this study from the prospective database of patients with IPN at a large Chinese tertiary hospital and attempted to identify the impacts of *Escherichia coli* infection on the outcomes of patients with IPN.

## Methods

### Patients

**Figure 1:**
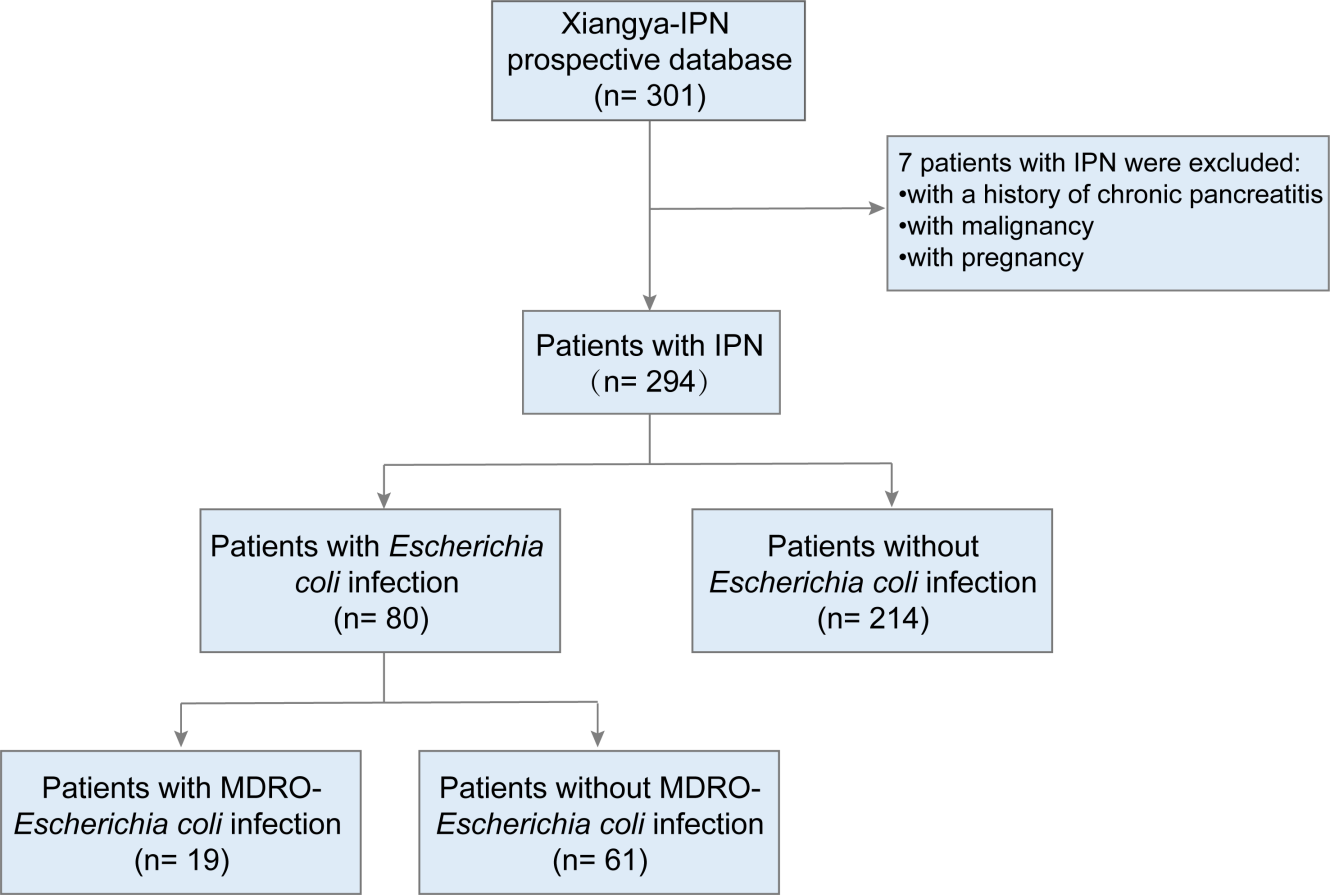
Flow diagram for patient inclusion.

A total of 294 patients (aged ≥ 18 years) with IPN, who were admitted to Xiangya Hospital (a large tertiary care center) between January 2010 and April 2022, were included in this study. We excluded patients with malignancy, a history of chronic pancreatitis, or those who were pregnant. The flow chart for patients is described in **Figure 1**. The follow-up period for the patients lasted 90 days from the day of discharge. The following data were collected from the prospectively institutional database of IPN (Shen et al., 2019b, Shen et al., 2021): clinical, radiological, microbiological characteristics, surgical management, and follow-up data.

All procedures performed in this study were conducted following the principles of the 7th revision of the Declaration of Helsinki. Ethical approval was obtained from the Institutional Review Board of Xiangya Hospital, and written informed consent was obtained from all patients or their representatives for the publication of data.

### Definitions and inclusion criteria

The diagnosis, classification, definitions, and managements associated with AP were based on the Revised Atlanta Classification (RAC) (Banks et al., 2013) and American Gastroenterological Association (AGA) guidelines (Crockett et al., 2018; Baron et al., 2020), and were also described in our previous studies (Ning et al., 2019; Shen et al., 2021; Li et al., 2022; Lin et al., 2022). Briefly, IPN was defined as the presence of gas bubbles within (peri)pancreatic necrosis on computed tomography (CT) and confirmed by a positive bacterial or fungal culture or Gram stain of (peri)pancreatic necrosis or fluid obtained from the first drainage procedure or the first necrosectomy. *Escherichia coli* infection was defined as an *Escherichia coli* culture from the pancreatic necrosis or fluid during the first necrosectomy or drainage. The severity of AP was defined per the RAC as follows: (1) mild acute pancreatitis (MAP): if there was no organ failure and no local or systemic complications. (2) moderately severe acute pancreatitis (MSAP): transient organ failure and/or local complications, and (3) severe acute pancreatitis (SAP): persistent organ failure (> 48 h) with a score of ≥ 2 using the modified Marshall scoring system.

For the surgical treatment of IPN, a step-up approach consisting of percutaneous catheter drainage (PCD), or endoscopic drainage was generally the first-line approach. If drainage did not improve clinically, subsequent minimally invasive or open pancreatic necrosectomy (OPN) would be performed to control the infection.

The MDROs were defined as organisms resistant to at least one antimicrobial agent in three or more antimicrobial categories (Magiorakos et al., 2012). MDROs-*Escherichia coli* included the extended-spectrum-β-lactamase-producing (ESBLp) and carbapenem-resistant Enterobacter cloacae.

Organ failure (OF) was defined as a score of 2 or more for the respiratory, cardiovascular, or renal organ systems using the modified Marshall scoring system as described in detail according to the RAC (Banks et al., 2013). Multiple organ failure (MOF) was defined as the failure of two or more organ systems.

Hemorrhage related to pancreatitis was defined as gastrointestinal bleeding, intraperitoneal or retroperitoneal bleeding. Pancreatic fistulas were characterized by elevated drain amylase (i.e., ≥3 times the upper limit of normal serum amylase concentration) of peripancreatic fluid obtained during drainage of debridement (Arvanitakis et al., 2018). Intestinal fistulas were defined as pathological communication connecting the intestinal tract with the necrotic cavity or the skin, including colonic fistula, duodenal fistula, and other intestinal fistulas (Shen et al., 2019a).

### Statistical analysis

Statistical analyses were performed using the SPSS version 26.0 software (SPSS, Inc., Chicago, IL, USA). Continuous variables were expressed as mean ± standard deviation or median and interquartile range as appropriate, and categorical variables were expressed as frequencies (n, %). Normal distributions were ascertained using the Shapiro–Wilk test before the parametric tests were performed. Categorical variables were analyzed using the chi-square test, and continuous variables were analyzed using the Mann–Whitney U and Student’s t-tests, as appropriate. Significant variables were included in the multivariate analysis, which was performed using logistic regression analysis, and odds ratios (ORs) were presented with 95% confidence intervals (CIs). The follow-up and survival data were calculated using the Kaplan–Meier method and performed through a log-rank test between-group comparison. Statistical significance was set at *P* < 0.05.

## Results

### Clinical characteristics of patients with and without *Escherichia coli* infection

**Figure 2:**
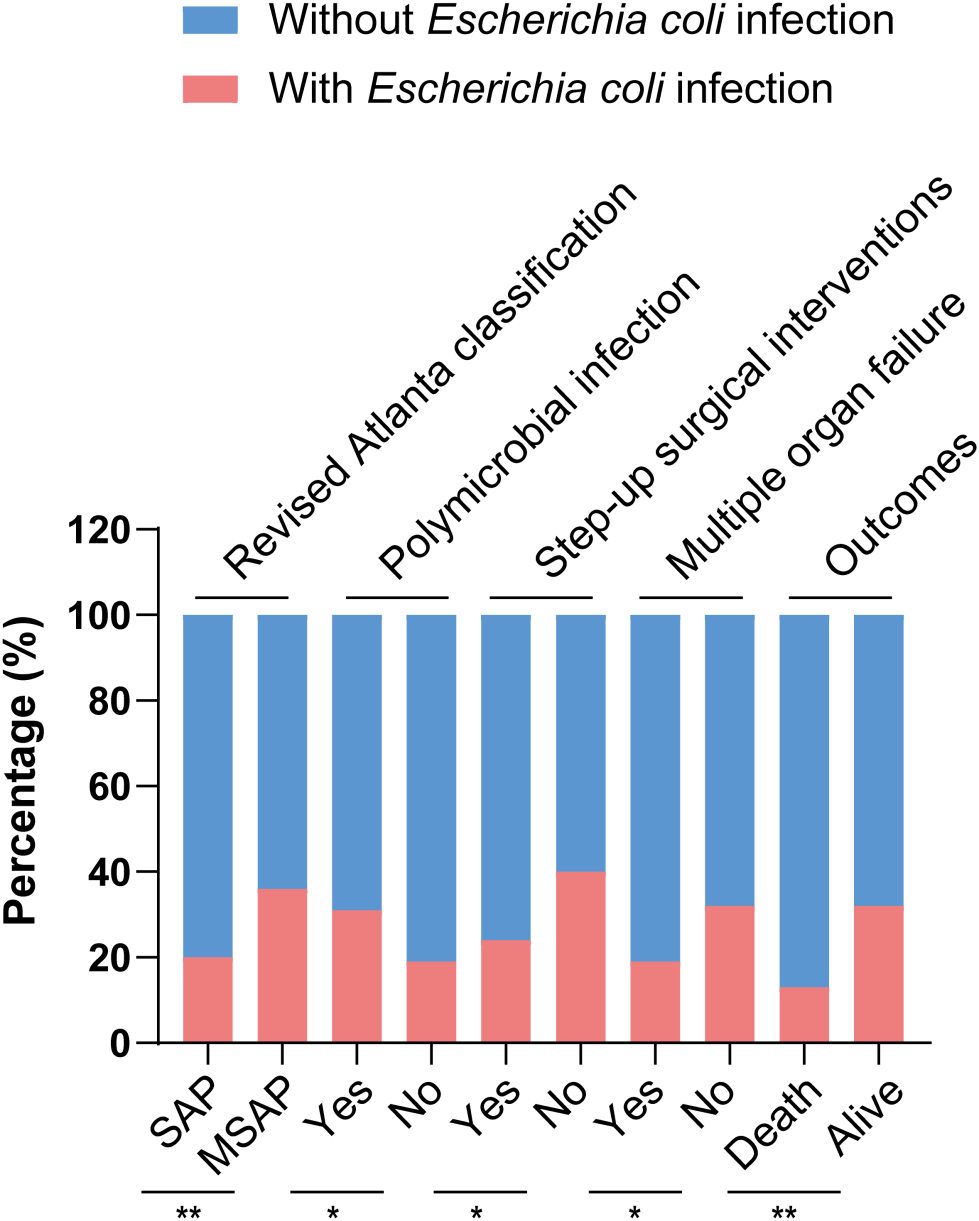
Comparisons of clinical characteristics between patients with and without *Escherichia coli* infection,.*p < 0.05 and **p < 0.01.

**Table 1** shows the baseline and clinical characteristics of patients with IPN. The cohort was divided into two groups based on the culture results from necrotic tissue or drainage fluid: the *Escherichia coli* infection group and the non-*Escherichia coli* infection group. Among the 294 patients with IPN, 80 (27.2%) and 214 (72.8%) had *Escherichia coli* and non-*Escherichia coli* infections, respectively. In the *Escherichia coli* infection group, there were 25 women (31.3%), with a mean age of 47.53 ± 13.26 years, and the most prevalent etiology was biliary (41.3%), followed by hyperlipidemia (38.8%), others (16.3%), and alcoholic (3.8%). There were no significant differences in baseline characteristics, including age, sex, and etiology, between the two groups. However, the SAP rate in the *Escherichia coli* infection group was significantly lower than that in the non-*Escherichia coli* infection group (42.5% vs. 61.7%, *P* = 0.003). Bloodstream infection occurrence had no significant differences between the two groups, while polymicrobial infection occurred more frequently in the *Escherichia coli* infection group than in the non-*Escherichia coli* infection group (77.5% vs. 65.0%, *P* = 0.04). The comparisons of the clinical characteristics between patients with and without *Escherichia coli* infection are shown in **Figure 2**. Among the 80 patients with *Escherichia coli* infection, the predominant MDROs were *Klebsiella pneumoniae* (33.8%), *Acinetobacter baumannii* (23.8%), and *Pseudomonas aeruginosa* (17.5%). The microorganism infection rates between the two groups showed no significant differences.

**Table 1:** Comparison of baseline and clinical characteristics among the four groups.

Characteristics	With *Escherichia coli* infection (n=80)			Without *Escherichia coli* infection (n=214)	*P* value^a^	*P* value^b^
	Total	Without MDRO-*Escherichia coli* infection (n=61)	With MDRO-*Escherichia coli* infection (n=19)			
Age, years (mean ± SD)	47.53±13.26	46.73 ± 12.80	48.89 ± 15.08	47.08 ± 12.02	0.982	0.484
Female, n (%)	25 (31.3)	15 (24.6)	10 (52.6)	61 (28.5)	0.645	0.021
Etiology, n (%)					0.804	0.177
Biliary	33 (41.3)	27 (44.3)	6 (31.6)	86 (40.2)		
Hypertriglyceridemia	31 (38.8)	20 (32.8)	11 (57.9)	94 (43.9)		
Alcoholic	3 (3.8)	2 (3.3)	1 (5.3)	7 (3.3)		
Others	13 (16.3)	12 (19.7)	1 (5.3)	27 (12.6)		
Revised Atlanta classification, n (%)					0.003	0.623
MSAP	46 (57.5)	36 (59.0)	10 (52.6)	82 (38.3)		
SAP	34 (42.5)	25 (41.0)	9 (47.4)	132 (61.7)		
Infection, n (%)						
Bloodstream infection	24 (30.0)	20 (32.8)	4 (21.1)	74 (34.6)	0.459	0.330
Polymicrobial infection	62 (77.5)	50 (82.0)	12 (63.2)	139 (65.0)	0.040	0.086
Microbiology profile of organisms, n (%)						
Klebsiella pneumoniae	27 (33.8)	19 (31.2)	8 (42.1)	82 (38.3)	0.471	0.378
Acinetobacter baumannii	19 (23.8)	16 (26.2)	3 (15.8)	62 (29.0)	0.372	0.350
Pseudomonas aeruginosa	14 (17.5)	11 (18.0)	3 (15.8)	27 (12.6)	0.282	0.822
Fungal infection	23 (28.8)	17 (27.9)	6 (31.6)	59 (27.6)	0.841	0.755

a Group with *Escherichia coli* infection vs. without *Escherichia coli* infection.

b Group Without MDRO-*Escherichia coli* infection vs. With MDRO-*Escherichia coli* infection.

SD, Standard deviations; MDRO, multidrug-resistant organism; MSAP, moderately severe acute pancreatitis; SAP, severe acute pancreatitis.

### Surgical interventions and outcomes between the patients with *Escherichia coli* infection and non-*Escherichia coli* infection

**Table 2** shows the surgical interventions, major complications, and outcomes. There were no significant difference in hospital stay after the first surgical intervention, the times of surgical interventions, ICU admission, length of hospital stay, and rates of major complications between IPN patients with and without *Escherichia coli* infection. In the study, the overall mortality rate was 25.5% (75/294). Mortality was significantly lower in patients with *Escherichia coli* infection (12.5%, 10/80) than in those without *Escherichia coli* infection (30.4%, 65/214, *p* = 0.002). In addition, patients with *Escherichia coli* infection needed fewer step-up surgical interventions (73.8% vs. 85.1%, *P* = 0.025) and had a lower rate of MOF (25.0% vs. 40.2%, *P* = 0.016) than those without *Escherichia coli* infection, although we found that patients in the *Escherichia coli* infection group had a longer hospital stay (median 82 vs. 78 days, *P* = 0.046). The comparisons on surgical interventions and outcomes between patients with and without *Escherichia coli* infection are shown in **Figure 2**.

**Table 2:** Comparison of patient outcomes among the four groups.

Characteristics	With *Escherichia coli* infection (n=80)			Without *Escherichia coli* infection (n=214)	*P*-value^a^	*P*-value^b^
	Total	Without MDRO-*Escherichia coli* infection (n=61)	With MDRO-*Escherichia coli* infection (n=19)			
Surgical interventions						
Time from onset to first surgical intervention, median days (IQR)	24 (15.0-41.0)	25 (15.0-39.0)	21 (12.0-44.0)	22 (14.0-33.0)	0.247	0.904
The hospital stays after the first surgical intervention (IQR)	36 (20.0-57.0)	37.5 (21.3-63.8)	26 (18.0-46.0)	33 (20.0-50.0)	0.206	0.109
Times need for interventions (IQR)	3 (2-5)	3 (2-5)	3 (2-5)	3 (2-4)	0.292	0.443
Step-up surgical interventions, n (%)	59 (73.8)	45 (73.8)	14 (73.7)	182 (85.1)	0.025	0.994
PCD alone, n (%)	17 (21.3)	12 (19.7)	5 (26.3)	42 (19.6)	0.757	0.536
Open necrosectomy, n(%)	28 (35.0)	22 (36.1)	6 (31.6)	52 (24.3)	0.067	0.720
Multiple organ failure (vs. single) , n (%)	20 (25.0)	15 (24.6)	5 (26.3)	86 (40.2)	0.016	0.879
ICU admission, n (%)	56 (70.0)	44 (72.1)	12 (63.2)	159 (74.3)	0.459	0.456
Hospital stays, days (IQR)	82 (67.8-135.0)	84 (68.5-137.0)	81 (65.0-114.0)	78 (56.0-107.5)	0.046	0.773
Major complications, n (%)						
Hemorrhage	17 (21.3)	11 (18.0)	6 (31.6)	50 (23.4)	0.700	0.208
Intestinal fistula	17 (21.3)	15 (24.6)	2 (10.5)	29 (13.6)	0.106	0.191
Pancreatic fistula	37 (46.3)	27 (44.3)	10 (52.6)	91 (42.5)	0.566	0.523
Mortality, n (%)	10 (12.5)	8 (13.1)	2 (10.5)	65 (30.4)	0.002	0.766

a Group with *Escherichia coli* infection vs. without *Escherichia coli* infection.

b Group Without MDRO-*Escherichia coli* infection vs. With MDRO-*Escherichia coli* infection.

IQR, interquartile range; PCD, percutaneous catheter drainage; ICU, intensive care unit.

### Clinical characteristics and outcomes between the patients with MDRO-*Escherichia coli* infection and non-MDRO *Escherichia coli* infection

**Tables 1**, **2** showed that the *Escherichia coli* infection group was further divided into MDRO-*Escherichia coli* infection and non-MDRO *Escherichia coli* infection. The baseline characteristics were equally distributed between the groups. There were no significant differences in clinical characteristics, including age, etiology, severity, the microbiological profile of organisms, surgical interventions, major complications, and mortality, between the two groups. In other words, the prognosis remained unchanged even if the *Escherichia coli* became multidrug-resistant. Interestingly, we found that the number of female patients was higher in the MDRO-*Escherichia coli* infection group than in patients with non-MDRO *Escherichia coli* infection (52.6% vs. 24.6%, *P* = 0.021).

### Predictors of mortality in patients with IPN

**Figure 3:**
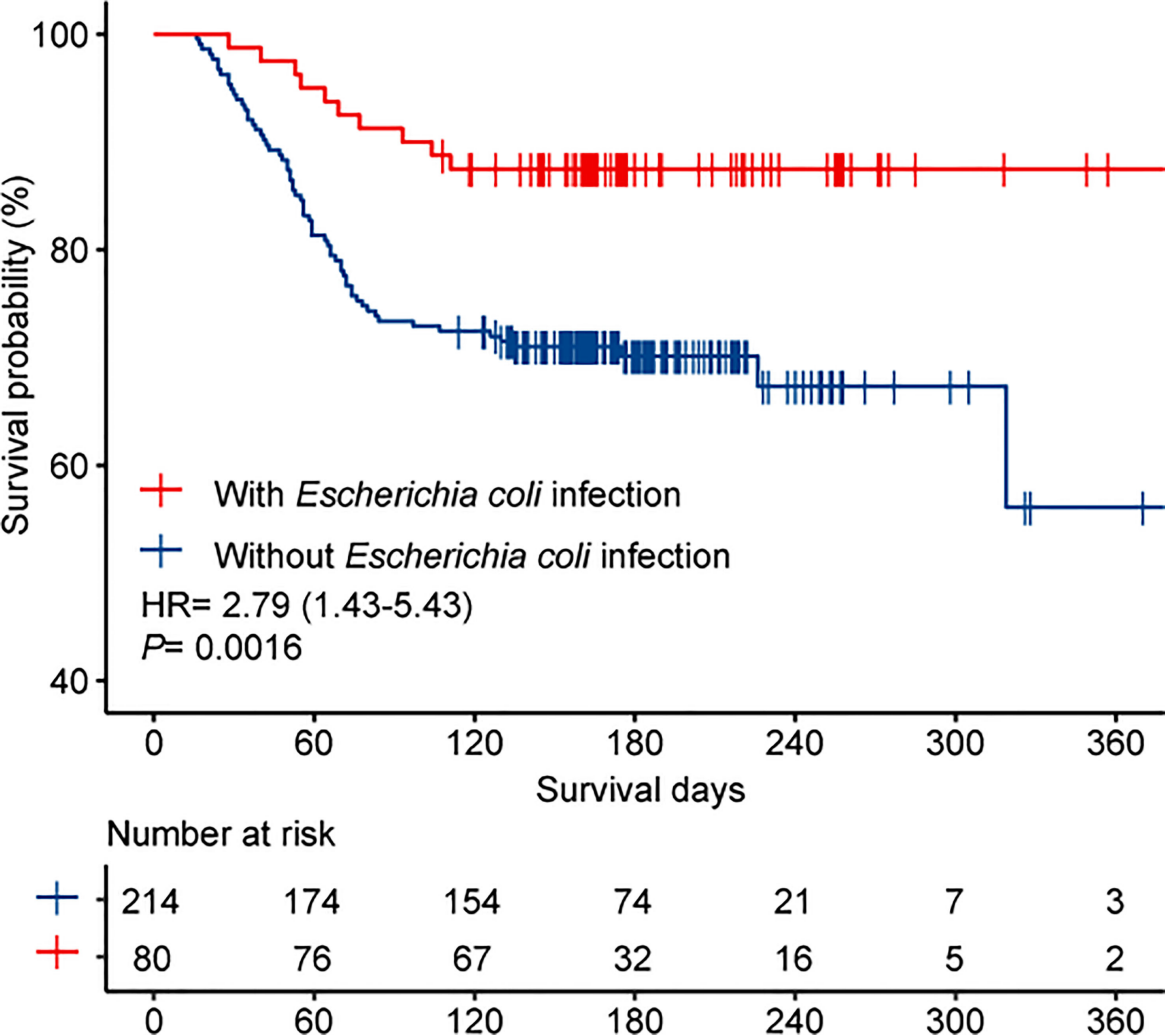
Kaplan-Meier survival estimate for patients with and without *Escherichia coli* infection.

The overall mortality rate in the entire cohort was 25.5% (75/294). **Table 3** shows the potential parameters that might predict death in patients with IPN. In the univariate analysis, SAP (*P*< 0.001), ICU admission (*P*< 0.001), MOF (*P* < 0.001), OPN (*P*= 0.002), hemorrhage (*P <*0.001), intestinal fistula (*P*=0.023), drainage fluid with *carbapenem-resistant Enterobacteriaceae* (CRE) infection (*P*= 0.003), drainage fluid with MDROs infection (*P* < 0.001), and bloodstream infection (*P* < 0.001) were associated with increased mortality. Interestingly, *Escherichia coli* infection was found to be associated with decreased mortality (OR, 0.327; 95% CI, 0.159–0.675; *P* = 0.003). In the multivariable analysis, MOF (OR, 6.197; 95% CI, 2.373–16.187; *P* < 0.001) and hemorrhage (OR, 3.485; 95% CI, 1.623–7.487; *P* = 0.001) were identified as independent predictors associated with higher mortality in patients with IPN, and the patients infected with *Escherichia coli* had significantly lower mortality than those who were not (OR, 0.302; 95% CI, 0.121–0.751; *P*=0.01). The Kaplan–Meier survival curves (**Figure 3**) showed that the survival outcome in the *Escherichia coli* infection group was significantly better than that in those without *Escherichia coli* infection (hazard ratio (HR) = 2.79 [1.43–5.43], *P* = 0.0016).

**Table 3:** Predictors of mortality in patients with IPN.

Variables	n (%)		Univariable analysis		Multivariable analysis	
	Died (n =75)	Survived (*n* =219)	OR (95% CI)	*P-*value	OR (95% CI)	*P-*value
SAP	68 (99.7)	98 (44.8)	11.994 (5.270–27.296)	<0.001	2.144 (0.594–7.744)	0.244
ICU admission	70 (93.3)	145 (66.2)	7.145 (2.765–18.465)	<0.001	0.680 (0.174–2.666)	0.581
Multiple organ failure (vs. single)	60 (80.0)	46 (21.0)	15.043 (7.833–28.890)	<0.001	6.197 (2.373–16.187)	<0.001
Open necrosectomy	31 (41.3)	49 (22.4)	2.444 (1.398–4.274)	0.002	1.92 (0.878–4.199)	0.102
Hemorrhage	40 (53.3)	27 (12.3)	8.127 (4.430–14.908)	<0.001	3.485 (1.623–7.487)	0.001
Intestinal fistula	18 (24.0)	28 (12.8)	2.154 (1.111–4.176)	0.023	1.706 (0.692–4.202)	0.246
Drainage fluid with CRE infection	32 (42.7)	54 (24.7)	2.274 (1.310–3.946)	0.003	1.531 (0.660–3.555)	0.321
Drainage fluid with MDROs infection	55 (73.3)	111 (50.7)	4.133 (2.264–7.545)	<0.001	0.715 (0.282–1.812)	0.480
Bloodstream infection	46 (61.3)	52 (23.7)	5.094 (2.912–8.912)	<0.001	1.921 (0.919–4.016)	0.083
With *Escherichia coli* infection	10 (13.3)	70 (32.0)	0.327 (0.159–0.675)	0.003	0.302 (0.121–0.751)	0.010

OR, odds ratio; CI, confidence interval; CRE, carbapenem-resistant enterobacteriaceae; MDROs, multidrug-resistant organisms.

## Discussion

To the best of our knowledge, this was the first and largest *post-hoc* study ever conducted in a tertiary hospital that evaluated the impacts of *Escherichia coli* infection on the outcomes of patients with IPN. This study demonstrated a significantly lower occurrence of SAP, fewer step-up surgical interventions, lower rate of MOF, and lower mortality in patients with IPN complicated with *Escherichia coli* infection. Additionally, this study revealed that MOF and hemorrhage were independent predictors of higher mortality in patients with IPN, and that the occurrence of *Escherichia coli* infection was a significant predictive factor for favorable prognosis in patients with IPN. These findings suggested that the prognosis of patients with IPN was significantly associated with the microbial culture results, which might help clinicians stratify patients based on microbial culture results.

IPN is considered a late-phase complication of AP, usually occurring two weeks after the onset of the disease. Although gut-derived bacteria cause most infections, the development of secondary necrotic collection infection is not fully understood (Liu et al., 2019; Thomas and Jobin, 2020). Significant gut barrier dysfunction has been observed in approximately 60% of patients with SAP (Wu et al., 2014). Gut barrier dysfunction and bacterial translocation from the gut are considered the central mechanisms underlying the development of peripancreatic infections (Li et al., 2020; Thomas and Jobin, 2020). *Escherichia coli* is one of the most frequently cultured microorganisms in the IPN and it is also an opportunistic pathogen derived from the gut (Mowbray et al., 2018). However, data regarding its role in IPN are still lacking. Li et al. (Li et al., 2013) conducted a prospective controlled study in patients with AP as revealed using 16S recombinant deoxyribonucleic acid (rDNA)-based molecular approaches, and they observed that translocated bacteria in patients with AP primarily consisted of opportunistic pathogens derived from the gut, including *Escherichia coli*, *Shigella flexneri*, *Enterobacteriaceae bacterium*, *Acinetobacter lwoffii*, *Bacillus coagulans*, and *Enterococcus faecium*. They also observed an association between the translocated bacterial spectrum of bacteremia and the severity of AP. Additionally, they revealed that more than half (60.4%) of the patients with AP encountered polymicrobial flora, which is consistent with our study’s results in that the polymicrobial infection rate was approximately 68.4% in IPN patients. An experimental animal study on the effects of *Escherichia coli* reported that *Escherichia coli* MG1655, a commensal belonging to *Escherichia*, increased intestinal injury and aggravated ANP in rats (Zheng et al., 2019), which seems to contradict our study’s conclusions. However, that study was concerned with the separate effects of *Escherichia coli*, whereas our this study focused on the role of *Escherichia coli* in polymicrobial infection of patients with IPN.

It has been established that many higher organisms harbor various complex microbial communities. Although interactions between microbial species in these communities can be cooperative, competitive interactions appear to be more common (Hibbing et al., 2010), which includes interference competition and exploitative competition. Interference competition is one of the modes of microbial competition, and it involves the disruption of quorum sensing (i.e., cell-cell communication in bacteria) by competitors (Raffatellu, 2018). Prior studies have reported that enteric bacteria such as *Escherichia coli* consume the quorum-sensing molecule autoinducer-2 so that inhibiting light production by V. harveyi (Xavier and Bassler, 2005). Another experimental study revealed that *Lactobacillus salivarius* UCC118, a strain of *Lactobacillus*, decreased dissemination from the gut to the spleen and liver of mice infected with the gram-positive pathogen *Listeria monocytogenes*, due to the benefits of the production of a bacteriocin termed Abp11849 (Corr et al., 2007), and bacteriocin production was also reported to mediate competition between *Enterococcus faecalis* strains of the gut in a follow-up study (Kommineni et al., 2015).

Furthermore, in some infectious and non-infectious colitis in mice, it has been demonstrated that *Enterobacteriaceae* compete for nutrients (Winter and Bäumler, 2014). In these settings, exploitative competition is essential determinants of which enteric strains predominate. For instance, intestinal clearance of the mouse gut pathogen *Citrobacter rodentium* can be achieved when a mouse is colonized with *Escherichia coli*. Similar to the growth of *Citrobacter rodentium*, *Escherichia coli* grows on carbohydrates (Kamada et al., 2012).

It is generally accepted that the gut microbiota can provide ‘colonization resistance’ by stimulating the host immune system and engaging in interference, and exploitative competition, which could provide a barrier against pathogens through colonization resistance (Kim et al., 2017). Similar competition mechanisms have been demonstrated at other body sites colonized by microbes, such as the skin and upper airways (Sassone-Corsi and Raffatellu, 2015). The results of this study showed a lower occurrence of several pathogenic bacteria in patients with IPN and *Escherichia coli* infection than in those without *Escherichia coli* infection, including *Klebsiella pneumoniae* infection (33.8% vs. 38.3%, *P* = 0.471) and *Acinetobacter baumannii* infection (23.8% vs. 29.0%, *P* = 0.372), although the difference was not significant. We speculated that *Escherichia coli* infection improved the outcomes of patients with IPN by colonization resistance and by conducting its competitive inhibition effect with other pathogenic bacteria of greater virulence.

Nevertheless, further studies, such as prospective studies with larger samples and animal experiments, are required to confirm the role of *Escherichia coli* infection in the disease process of IPN. Interestingly, in the present study, the prognosis remained unchanged even if the *Escherichia coli* infection became multidrug-resistant. There was no significant difference in the clinical outcomes of patients with IPN between the MDRO-*Escherichia coli* infection group and the non-MDRO-*Escherichia coli* infection group. This study demonstrated for the first time, to our knowledge, that females are more likely to develop the MDRO-*Escherichia coli* infection than males, but this conclusion requires verification in a larger sample size.

This study had some limitations. As the study was performed in a single center with a multidisciplinary team of specialists, it would be prudent to extrapolate the results, which may not apply to every institution. Additionally, despite this series representing one of the largest published to date addressing IPN with *Escherichia coli* infection, the sample size was still relatively small. Therefore, large-scale, multicenter, and prospective studies are needed to provide more precise conclusions and to reduce potential confounding results.

## Conclusions

This study showed a significantly lower occurrence of SAP, more frequent occurrence of polymicrobial infection, fewer step-up surgical interventions, lower rate of MOF, and lower mortality in IPN patients with *Escherichia coli* infection than in those without *Escherichia coli* infection. Additionally, this study revealed that MOF and hemorrhage were independent predictors of higher mortality in patients with IPN and that *Escherichia coli* infection was a significant factor associated with decreased mortality. These findings have vital implications for future antibiotic regimens in patients with IPN. However, more research is required to understand how opportunistic microbes compete with pathogens in the IPN and investigate bacterial metabolites with antimicrobial functions. As more is learned about specific mechanisms of microbial competition in the host, this knowledge can be leveraged to identify and deploy new opportunistic microbes and introduce these specific microbes or their metabolites to inhibit pathogenic strains, thus improving the prognosis.

## Data availability statement

The raw data supporting the conclusions of this article will be made available by the authors, without undue reservation.

## Ethics statement

The studies involving human participants were reviewed and approved by Institutional Review Board of Xiangya Hospital. The patients/participants provided their written informed consent to participate in this study.

## Author contributions

HH conceived the study. HH, JP, DS and GH participated in the study design. CN, QW, JL, CL, ZS, LC and SZ collected the data. HH and DS performed the statistical analyses. HH drafted the manuscript. DS and GH edited and checked the manuscript. All authors contributed to the article and approved the submitted version.
